# Clinical application of glucagon-like peptide-1 receptor agonists in cardiovascular disease: lessons from recent clinical cardiovascular outcomes trials

**DOI:** 10.1186/s12933-018-0731-y

**Published:** 2018-06-12

**Authors:** Atsushi Tanaka, Koichi Node

**Affiliations:** Department of Cardiovascular Medicine, Saga University, 5-1-1 Nabeshima, Saga, 849-8501 Japan

**Keywords:** Type 2 diabetes, Cardiovascular outcomes trial, Glucagon-like peptide-1 receptor agonist, Sodium-glucose cotransporter 2 inhibitor, Major adverse cardiovascular event

## Abstract

Recent clinical trials investigating cardiovascular (CV) safety of newer antidiabetic agents have been rapidly and largely changing the landscape of diabetes care and providing highly important clinical information on decision-making regarding the choice of antidiabetic agents. Similar to the sodium-glucose cotransporter 2 (SGLT2) inhibitors, some glucagon-like peptide-1 receptor agonists (GLP-1RAs) have also demonstrated a marked risk reduction in major adverse CV events (MACE) in patients with type 2 diabetes at high risk of CV events. However, the two classes of agents differ largely in their pharmacological modes of action on glucose-lowering and CV parameters. Furthermore, CV benefits on individual components of MACE and other outcomes, including heart failure (HF), appear to differ partly between the two classes. Specifically, improvement of overall CV outcomes was likely driven by reduction in HF-related events in trials investigating SGLT2 inhibitors, and by reduction in atherosclerotic events in those investigating GLP-1RAs. This difference in CV benefit observed in the trials has important clinical implications regarding how to use the two classes of agents and how to identify suitable patients to obtain the best benefit from each class during routine diabetes care, possibly leading to a treatment plan tailored to an individual patient’s CV risk and clinical condition. At this stage, however, we cardiologists may overlook such differences and may be unfamiliar with GLP-1RAs specifically. Herein, we highlight the potential benefits of GLP-1RAs on CV parameters observed in recent CV outcomes trials and further discuss clinical application of GLP-1RAs in CV medicine.

Health authority regulations in the US and Europe require pharmaceutical companies to conduct a pre-approval trial showing at least non-inferiority of cardiovascular (CV) safety compared with placebo treatment, for any new therapy to treat type 2 diabetes (T2D), prior to granting market approval. Following the momentous CV outcomes trial with sodium-glucose cotransporter 2 (SGLT2) inhibitor (empagliflozin) [1], two CV outcomes trials with glucagon-like peptide-1 receptor agonists (GLP-1RAs) have also demonstrated superior CV benefits compared with placebo [2, 3]. Furthermore, CV benefits have since been demonstrated for another SGLT2 inhibitor in the more recent CANVAS program [4]. These trials highlight the favorable effects of each new class of T2D therapy on CV parameters, seemingly resulting in a large paradigm shift in diabetes care and impact on decision-making in the selection of antidiabetic agents to improve prognosis [5, 6].

The two drug classes (SGLT2 inhibitors and GLP-1RAs) differ largely in their pharmacological actions. Compared with orally administered SGLT2 inhibitors, GLP-1RAs need to be subcutaneously injected, which appears to be a somewhat hurdle for patients to accept the therapy. However, given the superior CV benefits of GLP-1RAs, which differ somewhat from those attributed to SGLT2 inhibitors, we cardiologists also need to consider the CV benefits of GLP-1RAs and become more comfortable prescribing the agents for appropriate patients who may benefit the most from them. In this commentary, we summarize the potential effects of GLP-1RAs on CV parameters based on results from recent CV outcomes trials, and further discuss the clinical application of the agents in CV medicine.

Detailed biological characteristics of GLP-1 and proposed modes of action of GLP-1RAs on the metabolic and CV systems have been investigated in preclinical and clinical studies and summarized elsewhere [7–12]. In the LEADER (liraglutide) and SUSTAIN-6 (semaglutide) trials [2, 3], the GLP-1RAs rapidly and greatly decreased HbA_1c_ levels compared to placebo, which may have partly contributed to the increased incidence of diabetic retinopathy complications observed in SUSTAIN-6 (HR: 1.76 [95% CI 1.11, 2.78]). This suggests that appropriate management of diabetic retinopathy should be further required, especially when using semaglutide. On the other hand, the rates of occurrence of hypoglycemia in the active drug groups were similar (or even lower) to those of the placebo groups. Similar to the SGLT2 inhibitors [1, 4, 13], GLP-1RAs also mildly reduced systolic and diastolic blood pressure in the trials. Moreover, GLP-1RAs also modestly increased heart rate, although the exact mechanisms are still unclear. Similarly, body weight loss associated with GLP-1RAs was evident in the trials. The mechanism underlying body weight loss associated with SGLT2 inhibitors was speculated to be mainly due to plasma volume contraction and body fat mass reduction [14, 15], while body weight loss associated with GLP-1RAs appears to be at least in part due to a central effect. Interestingly, GLP-1RAs are known to reduce appetite and food intake partly via appetite-inhibitory neurotransmission signaling within the arcuate nucleus [16, 17], possibly resulting in subsequent improvements in insulin resistance and serum lipid profiles (e.g., reduction in triglyceride levels). Furthermore, decreases in body weight associated with GLP-1RAs differed between the CV outcomes trials, with mean body weight decreases from baseline of 2.3 kg at 36 months in the LEADER trial (median dose of liraglutide: 1.78 mg once-daily) and 3.6 and 4.9 kg at 104 weeks in the SUSTAIN-6 trial (for 0.5 and 1.0 mg once-weekly, respectively). This may indicate that the effect of semaglutide on body weight reduction is relatively rapid-acting and dose-dependent, possibly resulting from the higher albumin affinity of semaglutide compared with liraglutide [18]. Taken together, the metabolic modifications associated with GLP-1RA treatment appear to have a favorable impact on CV systems, such as vascular function [19, 20], subsequently leading to anti-atherosclerotic effects in T2D patients. In an experimental study, liraglutide treatment could also reduce vascular inflammation and suppress neointimal hyperplasia via improvement of nitric oxide bioavailability [21].

Although CV outcomes trials with GLP-1RAs (LEADER and SUSTAIN-6) and SGLT2 inhibitors (EMPA-REG OUTCOME and CANVAS) showed significantly reduced occurrences of the pre-specified primary composite endpoint comprised of CV death, non-fatal myocardial infarction, and non-fatal stroke in T2D patients at high risk of CV events, the treatment effects on the individual components of the primary composite endpoint differed between drug classes, and even between different members of the same class [22, 23]. While a consistent reduction in hospitalization for heart failure (HF) was confirmed in the CV outcomes trials with SGLT2 inhibitors, the effect of GLP-1RAs on hospitalization for HF was neutral [24]. This inconsistency between the drug classes may, in part, be caused by the presence or absence of diuretic action and increase in heart rate, both of which generally have a large impact on HF management. Furthermore, a previous randomized trial with liraglutide demonstrated that use of liraglutide in T2D patients with advanced HF and reduced left ventricular ejection fraction was associated with a numerically increased risk of death or rehospitalization for HF [25]. Atherosclerotic CV events (myocardial infarction and stroke) were totally and numerically prevented in LEADER and SUSTAIN-6. In contrast, both GLP-1RAs and SGLT2 inhibitors are known to have protective effects on renal function and improved renal outcomes were observed in CV outcomes trials [1, 3, 4, 26, 27]. Taken together, in addition to some common benefits for CV systems, it is likely that GLP-1RAs improved overall CV outcomes mainly by reduction in atherosclerotic events, whereas SGLT2 inhibitors improved CV outcomes mainly via prevention of HF-related outcomes (Fig. 1).

**Fig. 1:**
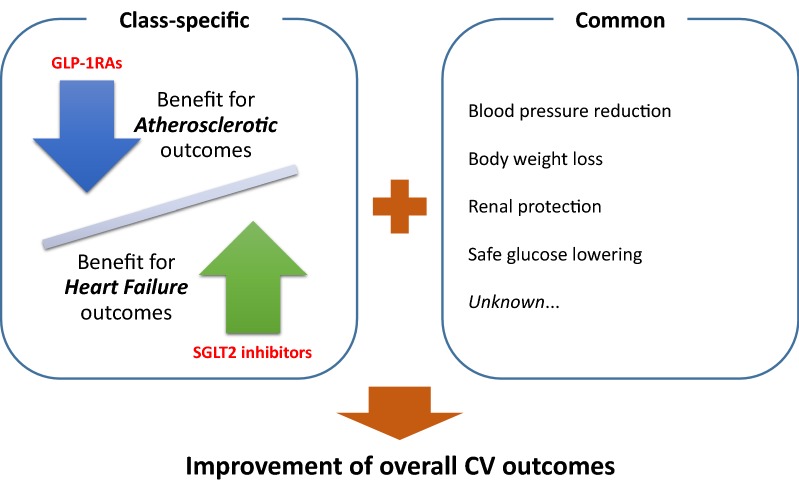
Potential effects of GLP-1RAs and SGLT2 inhibitors in CV outcomes trials. Based on the results from recent CV outcomes trials with GLP-1RAs and SGLT2 inhibitors, in addition to some common favorable CV effects associated with both classes, different class-specific effects (GLP-1RAs: benefit for risk of atherosclerotic outcomes, SGLT2 inhibitors: benefit for risk of HF outcomes) largely contributed to the overall improvement of CV outcomes. *CV* cardiovascular, *GLP*-*1RA* glucagon-like peptide-1 receptor agonist, *SGLT*2 sodium-glucose cotransporter 2

We also considered differences in trial designs and results among the CV outcomes trials with GLP-1RAs (Table 1). To date, four trials have been completed, and two trials are ongoing. Among the four completed trials, two (LEADER and SUSTAIN-6) demonstrated significant risk reduction in MACE [2, 3], and one (EXSCEL) demonstrated a numerically reduced risk of MACE [28]. In SUSTAIN-6, semaglutide treatment reduced the risk of MACE by 26% in a short follow-up duration (median 2.1 years). In contrast, in the ELIXA trial (once-daily lixisenatide) [29], neither an increase nor a decrease in MACE and the individual component CV events was observed during the follow-up duration (median 2.1 years). This may suggest the possibility that the short-acting nature of GLP-1 activation enhancement was insufficient to reduce the occurrence of CV events, beyond improved glycemic metabolism. Compared to the other trials investigating GLP-1RAs, the ELIXA trial included T2D patients with recent acute coronary syndrome (ACS), suggesting that perhaps their CV risk was too severe to demonstrate a simple decline in CV events via short-term pharmacological intervention. Furthermore, the patients in the trial had received higher rates of statin treatment (> 90%), and their levels of HbA_1c_ were lower (< 8.0%) than those of patients in the other trials. Such differences in trial designs and medical history of the patient populations may be a major determinant of the discrepancy in the results, although the exact reasons are currently unclear. In the more recent EXSCEL trial (once-weekly exenatide), all-cause mortality was statistically decreased by 14%, and CV death was numerically decreased by 12% [28]. Such outcomes were also significantly reduced by liraglutide treatment in the LEADER trial, but were not significantly reduced in the SUSTAIN-6 trial [2]. Focusing on the differences between the two trials, the LEADER trial included a higher rate (> 80%) of patients receiving secondary prevention of CV events, while the rate was 60% in the SUSTAIN-6 trial. Interestingly, among the CV outcomes trials with SGLT2 inhibitors, the EMPA REG OUTCOME trial, in which almost all patients had a history of CV events at baseline, showed marked reduction in all-cause mortality and CV death [1]. On the other hand, the CANVAS program, in which one-third of patients had no prior history of CV events, demonstrated no significant risk reduction in such outcomes [4]. We therefore speculate that the reduction in risk of all-cause mortality and CV death following pharmacological intervention depends, at least in part, on history of CV events at baseline. In the EXSCEL trial, > 70% of patients had a previous history of CV events, which was higher than in the SUSTAIN-6 and CANVAS trials. Moreover, a subgroup analysis revealed that exenatide treatment was numerically better at preventing MACE in patients with a previous history of CV events relative to those without, although there was no statistically significant interaction of the treatment effect across the subgroups [28]. A similar trend was also demonstrated in a subgroup analysis of the CANVAS trial [30]. Finally, the treatment effect of GLP-1RAs on the hospitalization for HF was consistently neutral across the trials [2, 3, 28, 29]. Thus, these results suggest that GLP-1RAs exhibit superior preventive effects on atherosclerotic CV events and can improve CV prognosis in T2D patients with a previous history of CV events (except ACS), and that such treatment effects could become apparent by, for example, comparing the results of CV outcomes trials with another class of incretin-based agents, namely dipeptidyl peptidase-4 (DPP-4) inhibitors [31–33].

**Table 1:** Comparion of recent CV outcomes trials completed with GLP-1RAs and SGLT2 inhibitors

	GLP-1RA				SGLT2 inhibitor	
	Once-daily		Once-weekly			
	ELIXA (lixisenatide)	LEADER (liraglutide)	SUSTAIN-6 (semaglutide)	EXSCEL (exenatide)	EMPA-REG OUTCOME (empagliflozin)	CANVAS^a^ (canagliflozin)
Patient number	6058	9340	3297	14,752	7020	10,142
Median follow-up duration (years)	2.1	3.8	2.1	3.2	3.1	2.4
Key eligibility	T2D with recent ACS	T2D with high CV risk	T2D with high CV risk	T2D with high CV risk	T2D with previous CV disease	T2D with high CV risk
Prior CV disease (%)	100	81	60	73	99	66
Mean baseline HbA1c (%)	7.7	8.7	8.7	8.0	8.1	8.2
Metformin use (%)	66	76	73	77	74	77
Statin use (%)	93	72	73	74	77	75
RAAS inhibitor use (%)	85	83	84	80	81	80
Outcomes (HR [95% CI])						
MACE^b^	1.02 [0.89–1.17]	*0.87 [0.78–0.97]*	*0.74 [0.58–0.95]*	0.91 [0.83–1.00]	*0.86 [0.74–0.99]*	*0.86 [0.75* *–* *0.97]*
CV death	0.98 [0.78–1.22]	*0.78 [0.66–0.93]*	0.98 [0.65–1.48]	0.88 [0.76–1.02]	*0.62 [0.49* *–* *0.77]*	0.87 [0.72–1.06]
Myocardial infarction	1.03 [0.87–1.22]	0.86 [0.73–1.00]	0.74 [0.51–1.08]	0.97 [0.85–1.10]	0.87 [0.70–1.09]	0.85 [0.69–1.05]
Stroke	1.12 [0.79–1.58]	0.86 [0.71–1.06]	*0.61 [0.38–0.99]*	0.85 [0.70–1.03]	1.18 [0.89–1.56]	0.90 [0.71–1.15]
All-cause death	0.94 [0.78–1.13]	*0.85 [0.74–0.97]*	1.05 [0.74–1.50]	*0.86 [0.77–0.97]*	*0.68 [0.57* *–* *0.82]*	0.87 [0.74–1.01]
Hospitalization for heart failure	0.96 [075–1.23]	0.87 [0.73–1.05]	1.11 [0.77–1.61]	0.94 [0.78–1.13]	*0.65 [0.50* *–* *0.85]*	*0.67 [0.52* *–* *0.87]*
Composite renal outcomes	–	*0.78 [0.67–0.92]*	*0.64 [0.46–0.88]*	–	*0.61 [0.53* *–* *0.70]*	*0.60 [0.47–0.77]*

Italic values indicate significance of p value (p < 0.05)

*ACS* acute coronary syndrome, *CI* confidence interval, *CV* cardiovascular, *GLP*-*1RA* glucagon-like peptide-1 receptor agonist, *MACE* major adverse cardiovascular events, *HR* hazard ratio, *RAAS* renin–angiotensin–aldosterone system, *SGLT2* sodium-glucose cotransporter 2, *T2D* type 2 diabetes

^a^Pooled data from CANVAS and CANVAS-R

^b^4-point MACE in the ELIXA trial and 3-point MACE in the other trials

Assessment of the reasons for the mismatch between the results of CV outcomes trials with GLP-1RAs and DPP-4 inhibitors remains a challenge. Both are incretin-based glucose-lowering agents that act via enhancement of GLP-1 activity; however, CV outcomes trials with these agents showed apparently different treatment effects on CV outcomes. In contrast to the CV outcomes trials with GLP-1RAs, those with DPP-4 inhibitors demonstrated consistent non-inferiority in both the primary composite outcome and individual components compared to placebo, and even showed some unexpected increases in risk of hospitalization for HF, acute pancreatitis, and hypoglycemia [31–34]. Although the exact mechanisms by which both classes of agents exert their inconsistent effects on CV safety remain unknown, there are apparently distinct modes of action resulting in different enhancement of GLP-1 levels and non-glycemic effects [35]. Dr. Packer recently proposed an intriguing hypothesis that DPP-4 inhibitors potentiate some endogenous peptides, such as stromal cell-derived factor-1, in addition to activation of GLP-1, and that unfavorable effects of those peptides on CV parameters (e.g., inflammation, fibrosis, plaque growth, and sympathetic activation) could prevail against the CV protective effects of GLP-1, possibly leading to worsened CV complications and HF [36, 37]. Thus, based on the results of CV outcomes trials with incretin-based agents, clinical priority of GLP-1RAs may be accentuated when choosing antidiabetic agents, at least for T2D patients at higher risk of CV events. Furthermore, interestingly a recent report from Japan demonstrated that dulaglutide 0.75 mg once weekly was a cost-effective therapy, compared to insulin glargine [38].

In summary, recent CV outcomes trials with GLP-1RAs have shown promising benefits in CV care of T2D patients, and GLP-1RAs have been accordingly listed as a second line therapy in T2D patients with established atherosclerotic CV disease, alongside SGLT2 inhibitors [5, 6]. As the pharmacological action and impact on CV parameters interpreted from the CV outcomes trials seemingly differed between both classes of glucose-lowering agents, they should be used accordingly, that is, after taking into consideration a patient’s needs and drug tolerability. Further studies are also needed to assess mechanistic insights into GLP-1RAs on CV parameters and clinical safety of GLP-1RAs especially in elderly populations.

## Abbreviations

ACS: acute coronary syndrome
CV: cardiovascular
GLP-1RA: glucagon-like peptide-1 receptor agonist
DPP-4: dipeptidyl peptidase-4
HF: heart failure
MACE: major adverse CV event
SGLT2: sodium-glucose cotransporter 2
T2D: type 2 diabetes
